# 3-Ethyl 5-methyl 4-(2,3-dichloro­phen­yl)-2,6-dimethyl­pyridine-3,5-dicarboxyl­ate

**DOI:** 10.1107/S1600536810003235

**Published:** 2010-02-06

**Authors:** Jing Luo, Hui Chen, Qiao-Feng Wang, Hai-Jing Liu

**Affiliations:** a ShaanXi Institute For Food And Drug Control, Zhuque Road 431, 710061 Xi-An, People’s Republic of China; b Department of Chemistry, School of Pharmacy, Fourth Military Medical University, Changle West Road 17, 710032 Xi-An, People’s Republic of China

## Abstract

In the title compound, C_18_H_17_Cl_2_NO_4_, an oxidation product of felodipine, the dihedral angle between the benzene and pyridine rings is 75.3 (4)°. The crystal structure is stabilized by intermolecular C—H⋯O interactions.

## Related literature

For related structures, see: Baranda *et al.* (2004 ▶); Che *et al.* (2004 ▶); Won *et al.* (2005 ▶); Xu *et al.* (1995 ▶). For felodipine derivatives as calcium channel blockers with vasodilator properties, see: Ferrari *et al.* (2005 ▶); Qin *et al.* (1995 ▶); Marciniec *et al.* (2002 ▶).
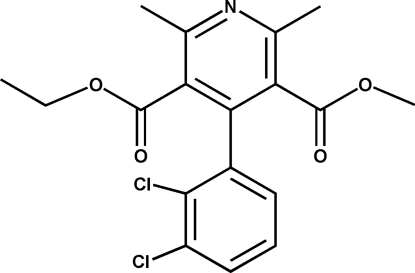


## Experimental

### 

#### Crystal data

C_18_H_17_Cl_2_NO_4_
*M* *_r_* = 382.23
Orthorhombic, 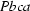

*a* = 14.3179 (6) Å
*b* = 15.5045 (7) Å
*c* = 16.9664 (8) Å
*V* = 3766.4 (3) Å^3^
*Z* = 8
Mo *K*α radiation
μ = 0.37 mm^−1^
*T* = 296 K
0.12 × 0.09 × 0.08 mm

#### Data collection

Bruker APEXII CCD diffractometer
Absorption correction: multi-scan (*SADABS*; Bruker, 2001 ▶) *T*_min_ = 0.957, *T*_max_ = 0.971
21810 measured reflections
4268 independent reflections
2268 reflections with *I* > 2σ(*I*)
*R*_int_ = 0.054

#### Refinement

*R*[*F* ^2^ > 2σ(*F* ^2^)] = 0.056
*wR*(*F* ^2^) = 0.153
*S* = 1.00
4268 reflections
230 parameters
H-atom parameters constrained
Δρ_max_ = 0.36 e Å^−3^
Δρ_min_ = −0.26 e Å^−3^

### 

Data collection: *APEX2* (Bruker, 2004 ▶); cell refinement: *SAINT-Plus* (Bruker, 2001 ▶); data reduction: *SAINT-Plus*; program(s) used to solve structure: *SHELXS97* (Sheldrick, 2008 ▶); program(s) used to refine structure: *SHELXL97* (Sheldrick, 2008 ▶); molecular graphics: *SHELXTL* (Sheldrick, 2008 ▶); software used to prepare material for publication: *SHELXTL*.

## Supplementary Material

Crystal structure: contains datablocks I, global. DOI: 10.1107/S1600536810003235/pk2222sup1.cif

Structure factors: contains datablocks I. DOI: 10.1107/S1600536810003235/pk2222Isup2.hkl

Additional supplementary materials:  crystallographic information; 3D view; checkCIF report

## Figures and Tables

**Table 1:** Hydrogen-bond geometry (Å, °)

*D*—H⋯*A*	*D*—H	H⋯*A*	*D*⋯*A*	*D*—H⋯*A*
C5—H5⋯O1^i^	0.93	2.55	3.303 (4)	139
C16—H16*C*⋯O1^ii^	0.96	2.67	3.328 (5)	126
C17—H17*B*⋯O4^iii^	0.97	2.55	3.247 (4)	129

Symmetry codes: (i) 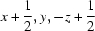; (ii) 
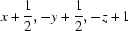
; (iii) 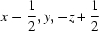.

**Fig. 1.**
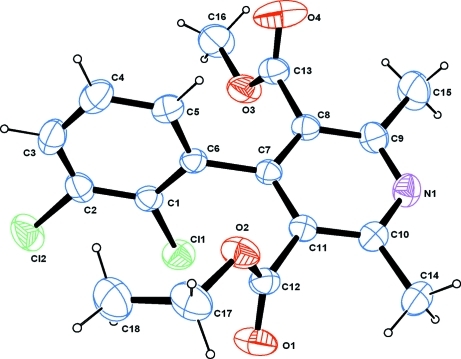
Molecular structure of the title compound (I). Displacement ellipsoids are drawn at the 30% probability level.

## supplementary crystallographic information

### Comment

Felodipine is a calcium channel blocker with vasodilator properties. It is used in several commercial preparations for treatment of hypertension (Marciniec *et al.*,  2002). It has been the subject of many analytical chemical investigations.  Regulations on the purity profile of bulk drug substances require determination of levels of impurities such as the title compound, which is an oxidation product of felodipine.

The molecular structure is shown in Fig 1. The dihedral angle between the phenyl ring and the pyridine ring is 75.3 (4)°.  The crystal structure is stabilized by van der Waals interactions.

### Experimental

A mixture of 5 g felodipine and 50 mL 50% sulfuric acid (50%, *v*/*v*) was heated under reflux for 30 min, then cooled to room temperature and saturated sodium hydroxide was added slowly until the solution was neutral. The mixture was then extracted with 3×100 ml CHCl_3_ and the combined organic layers were dried over Na_2_SO4 before solvent removal. The product was purified by chromatography on silica gel with 1:1 EtOAc / hexanes as eluent.

### Refinement

H-atoms were included in calculated positions and treated as riding atoms: C-H = 0.92—0.97 Å with Uiso(H) = 1.2—1.5 Ueq(C)

### Crystal data

**Table:**

C_18_H_17_Cl_2_NO_4_	*F*(000) = 1584
*M**_r_* = 382.23	*D*_x_ = 1.348 Mg m^−^^3^
Orthorhombic, *P**b**c**a*	Mo *K*α radiation, λ = 0.71073 Å
Hall symbol: -P 2ac 2ab	Cell parameters from 2869 reflections
*a* = 14.3179 (6) Å	θ = 2.4–20.2°
*b* = 15.5045 (7) Å	µ = 0.37 mm^−^^1^
*c* = 16.9664 (8) Å	*T* = 296 K
*V* = 3766.4 (3) Å^3^	Block, colorless
*Z* = 8	0.12 × 0.09 × 0.08 mm

### Data collection

**Table:**

Bruker APEXII CCD diffractometer	4268 independent reflections
Radiation source: fine-focus sealed tube	2268 reflections with *I* > 2σ(*I*)
graphite	*R*_int_ = 0.054
φ and ω scans	θ_max_ = 27.5°, θ_min_ = 2.3°
Absorption correction: multi-scan (*SADABS*; Bruker, 2001)	*h* = −18→11
*T*_min_ = 0.957, *T*_max_ = 0.971	*k* = −19→18
21810 measured reflections	*l* = −21→21

### Refinement

**Table:**

Refinement on *F*^2^	Primary atom site location: structure-invariant direct methods
Least-squares matrix: full	Secondary atom site location: difference Fourier map
*R*[*F*^2^ > 2σ(*F*^2^)] = 0.056	Hydrogen site location: inferred from neighbouring sites
*wR*(*F*^2^) = 0.153	H-atom parameters constrained
*S* = 1.00	*w* = 1/[σ^2^(*F*_o_^2^) + (0.056*P*)^2^ + 2.0434*P*] where *P* = (*F*_o_^2^ + 2*F*_c_^2^)/3
4268 reflections	(Δ/σ)_max_ < 0.001
230 parameters	Δρ_max_ = 0.36 e Å^−^^3^
0 restraints	Δρ_min_ = −0.26 e Å^−^^3^

### Special details

**Table:**

Geometry. All e.s.d.'s (except the e.s.d. in the dihedral angle between two l.s. planes) are estimated using the full covariance matrix. The cell e.s.d.'s are taken into account individually in the estimation of e.s.d.'s in distances, angles and torsion angles; correlations between e.s.d.'s in cell parameters are only used when they are defined by crystal symmetry. An approximate (isotropic) treatment of cell e.s.d.'s is used for estimating e.s.d.'s involving l.s. planes.
Refinement. Refinement of *F*^2^ against ALL reflections. The weighted *R*-factor *wR* and goodness of fit *S* are based on *F*^2^, conventional *R*-factors *R* are based on *F*, with *F* set to zero for negative *F*^2^. The threshold expression of *F*^2^ > σ(*F*^2^) is used only for calculating *R*-factors(gt) *etc*. and is not relevant to the choice of reflections for refinement. *R*-factors based on *F*^2^ are statistically about twice as large as those based on *F*, and *R*- factors based on ALL data will be even larger.

### Fractional atomic coordinates and isotropic or equivalent isotropic displacement parameters (Å^2^)

**Table:**

	*x*	*y*	*z*	*U*_iso_*/*U*_eq_	
C1	0.6713 (2)	0.16190 (18)	0.34902 (17)	0.0502 (7)	
C2	0.6979 (2)	0.08675 (19)	0.30988 (19)	0.0616 (8)	
C3	0.7775 (3)	0.0859 (2)	0.2636 (2)	0.0718 (10)	
H3	0.7955	0.0357	0.2378	0.086*	
C4	0.8295 (2)	0.1596 (2)	0.2559 (2)	0.0715 (9)	
H4	0.8832	0.1591	0.2251	0.086*	
C5	0.8034 (2)	0.2339 (2)	0.29326 (18)	0.0584 (8)	
H5	0.8393	0.2834	0.2872	0.070*	
C6	0.72405 (18)	0.23629 (18)	0.33994 (16)	0.0462 (7)	
C7	0.69755 (18)	0.31818 (17)	0.38064 (16)	0.0448 (6)	
C8	0.74662 (19)	0.34553 (19)	0.44663 (16)	0.0504 (7)	
C9	0.7225 (2)	0.4235 (2)	0.48194 (18)	0.0608 (8)	
C10	0.6020 (2)	0.44520 (19)	0.39427 (18)	0.0542 (7)	
C11	0.62486 (18)	0.37031 (18)	0.35304 (16)	0.0460 (7)	
C12	0.5729 (2)	0.34657 (18)	0.28006 (18)	0.0519 (7)	
C13	0.8256 (2)	0.2937 (2)	0.47870 (17)	0.0594 (8)	
C14	0.5231 (2)	0.5037 (2)	0.3710 (2)	0.0731 (10)	
H14A	0.4807	0.5098	0.4145	0.110*	
H14B	0.4907	0.4796	0.3267	0.110*	
H14C	0.5476	0.5593	0.3570	0.110*	
C15	0.7767 (3)	0.4590 (3)	0.5511 (2)	0.0977 (13)	
H15A	0.8121	0.5084	0.5344	0.147*	
H15B	0.8183	0.4155	0.5707	0.147*	
H15C	0.7340	0.4757	0.5920	0.147*	
C16	0.8683 (3)	0.1748 (3)	0.5567 (3)	0.1154 (17)	
H16A	0.9022	0.1437	0.5170	0.173*	
H16B	0.8388	0.1347	0.5919	0.173*	
H16C	0.9106	0.2107	0.5858	0.173*	
C17	0.5886 (3)	0.3220 (3)	0.1420 (2)	0.0806 (11)	
H17A	0.6231	0.3485	0.0992	0.097*	
H17B	0.5241	0.3409	0.1383	0.097*	
C18	0.5928 (3)	0.2273 (3)	0.1341 (2)	0.0997 (14)	
H18A	0.6557	0.2081	0.1429	0.150*	
H18B	0.5734	0.2110	0.0821	0.150*	
H18C	0.5522	0.2012	0.1723	0.150*	
Cl1	0.57453 (6)	0.16217 (6)	0.40985 (6)	0.0735 (3)	
Cl2	0.63272 (8)	−0.00639 (6)	0.31910 (7)	0.0982 (4)	
N1	0.65082 (19)	0.47100 (17)	0.45730 (15)	0.0630 (7)	
O1	0.49233 (15)	0.32839 (17)	0.27743 (14)	0.0830 (8)	
O2	0.62809 (14)	0.35000 (15)	0.21758 (12)	0.0665 (6)	
O3	0.79723 (16)	0.22840 (16)	0.51927 (17)	0.0860 (8)	
O4	0.90563 (18)	0.3137 (2)	0.47082 (18)	0.1162 (12)	

### Atomic displacement parameters (Å^2^)

**Table:**

	*U*^11^	*U*^22^	*U*^33^	*U*^12^	*U*^13^	*U*^23^
C1	0.0476 (15)	0.0505 (17)	0.0526 (17)	0.0011 (14)	−0.0078 (13)	0.0058 (14)
C2	0.070 (2)	0.0493 (18)	0.065 (2)	0.0015 (16)	−0.0240 (17)	0.0022 (15)
C3	0.085 (3)	0.067 (2)	0.063 (2)	0.028 (2)	−0.0124 (19)	−0.0119 (18)
C4	0.068 (2)	0.086 (3)	0.061 (2)	0.019 (2)	0.0066 (17)	−0.0047 (19)
C5	0.0483 (17)	0.066 (2)	0.061 (2)	0.0025 (15)	0.0045 (15)	0.0006 (16)
C6	0.0410 (15)	0.0518 (16)	0.0458 (16)	0.0036 (13)	−0.0063 (12)	−0.0002 (13)
C7	0.0410 (15)	0.0482 (16)	0.0452 (16)	−0.0027 (13)	0.0025 (12)	0.0009 (12)
C8	0.0471 (15)	0.0592 (18)	0.0449 (16)	−0.0013 (14)	−0.0014 (14)	0.0008 (14)
C9	0.069 (2)	0.065 (2)	0.0484 (18)	−0.0016 (17)	−0.0028 (15)	−0.0051 (15)
C10	0.0550 (18)	0.0545 (18)	0.0532 (19)	0.0024 (14)	0.0056 (14)	0.0058 (14)
C11	0.0452 (15)	0.0486 (16)	0.0443 (16)	−0.0032 (13)	0.0005 (12)	0.0035 (13)
C12	0.0454 (17)	0.0539 (17)	0.0565 (19)	0.0012 (14)	−0.0054 (14)	0.0057 (14)
C13	0.054 (2)	0.079 (2)	0.0446 (17)	0.0017 (17)	−0.0055 (15)	−0.0050 (16)
C14	0.072 (2)	0.062 (2)	0.085 (3)	0.0173 (17)	−0.0004 (19)	−0.0003 (18)
C15	0.122 (3)	0.098 (3)	0.074 (3)	0.013 (3)	−0.034 (2)	−0.033 (2)
C16	0.099 (3)	0.072 (3)	0.175 (5)	0.014 (2)	−0.065 (3)	0.022 (3)
C17	0.088 (3)	0.103 (3)	0.051 (2)	−0.009 (2)	−0.0154 (18)	−0.0036 (19)
C18	0.113 (3)	0.103 (3)	0.083 (3)	0.000 (3)	−0.019 (2)	−0.022 (2)
Cl1	0.0591 (5)	0.0707 (6)	0.0907 (6)	−0.0108 (4)	0.0126 (4)	0.0090 (5)
Cl2	0.1146 (8)	0.0497 (5)	0.1304 (10)	−0.0090 (5)	−0.0369 (7)	0.0011 (5)
N1	0.0763 (18)	0.0573 (16)	0.0555 (17)	0.0072 (14)	−0.0004 (14)	−0.0067 (13)
O1	0.0480 (13)	0.125 (2)	0.0764 (17)	−0.0154 (14)	−0.0082 (11)	−0.0051 (15)
O2	0.0584 (13)	0.0900 (17)	0.0512 (13)	−0.0115 (11)	−0.0062 (10)	−0.0020 (11)
O3	0.0682 (15)	0.0638 (15)	0.126 (2)	−0.0021 (12)	−0.0318 (15)	0.0242 (15)
O4	0.0533 (16)	0.182 (3)	0.114 (2)	0.0082 (18)	−0.0036 (15)	0.057 (2)

### Geometric parameters (Å, °)

**Table:**

C1—C6	1.387 (4)	C12—O1	1.188 (3)
C1—C2	1.394 (4)	C12—O2	1.323 (3)
C1—Cl1	1.728 (3)	C13—O4	1.194 (4)
C2—C3	1.384 (5)	C13—O3	1.290 (4)
C2—Cl2	1.726 (3)	C14—H14A	0.9602
C3—C4	1.371 (5)	C14—H14B	0.9602
C3—H3	0.9300	C14—H14C	0.9602
C4—C5	1.367 (4)	C15—H15A	0.9601
C4—H4	0.9300	C15—H15B	0.9601
C5—C6	1.385 (4)	C15—H15C	0.9601
C5—H5	0.9300	C16—O3	1.459 (4)
C6—C7	1.494 (4)	C16—H16A	0.9600
C7—C11	1.398 (4)	C16—H16B	0.9600
C7—C8	1.388 (4)	C16—H16C	0.9600
C8—C9	1.393 (4)	C17—O2	1.467 (4)
C8—C13	1.490 (4)	C17—C18	1.476 (5)
C9—N1	1.331 (4)	C17—H17A	0.9700
C9—C15	1.510 (4)	C17—H17B	0.9700
C10—N1	1.339 (4)	C18—H18A	0.9600
C10—C11	1.395 (4)	C18—H18B	0.9600
C10—C14	1.501 (4)	C18—H18C	0.9600
C11—C12	1.491 (4)		
			
C6—C1—C2	119.6 (3)	O4—C13—O3	124.5 (3)
C6—C1—Cl1	120.1 (2)	O4—C13—C8	123.2 (3)
C2—C1—Cl1	120.4 (2)	O3—C13—C8	112.2 (3)
C3—C2—C1	120.2 (3)	C10—C14—H14A	109.5
C3—C2—Cl2	119.2 (3)	C10—C14—H14B	109.5
C1—C2—Cl2	120.6 (3)	H14A—C14—H14B	109.5
C4—C3—C2	119.6 (3)	C10—C14—H14C	109.5
C4—C3—H3	120.2	H14A—C14—H14C	109.5
C2—C3—H3	120.2	H14B—C14—H14C	109.5
C3—C4—C5	120.6 (3)	C9—C15—H15A	109.5
C3—C4—H4	119.7	C9—C15—H15B	109.5
C5—C4—H4	119.7	H15A—C15—H15B	109.5
C4—C5—C6	120.8 (3)	C9—C15—H15C	109.5
C4—C5—H5	119.6	H15A—C15—H15C	109.5
C6—C5—H5	119.6	H15B—C15—H15C	109.5
C5—C6—C1	119.2 (3)	O3—C16—H16A	109.5
C5—C6—C7	119.7 (3)	O3—C16—H16B	109.5
C1—C6—C7	121.1 (2)	H16A—C16—H16B	109.5
C11—C7—C8	118.1 (3)	O3—C16—H16C	109.5
C11—C7—C6	121.7 (2)	H16A—C16—H16C	109.5
C8—C7—C6	120.2 (2)	H16B—C16—H16C	109.5
C9—C8—C7	119.1 (3)	O2—C17—C18	110.9 (3)
C9—C8—C13	119.9 (3)	O2—C17—H17A	109.5
C7—C8—C13	120.9 (3)	C18—C17—H17A	109.5
N1—C9—C8	122.4 (3)	O2—C17—H17B	109.5
N1—C9—C15	116.0 (3)	C18—C17—H17B	109.4
C8—C9—C15	121.5 (3)	H17A—C17—H17B	108.0
N1—C10—C11	121.8 (3)	C17—C18—H18A	109.5
N1—C10—C14	114.9 (3)	C17—C18—H18B	109.5
C11—C10—C14	123.3 (3)	H18A—C18—H18B	109.5
C7—C11—C10	119.2 (3)	C17—C18—H18C	109.5
C7—C11—C12	120.5 (3)	H18A—C18—H18C	109.5
C10—C11—C12	120.3 (3)	H18B—C18—H18C	109.5
O1—C12—O2	124.0 (3)	C9—N1—C10	119.3 (3)
O1—C12—C11	125.1 (3)	C12—O2—C17	117.3 (2)
O2—C12—C11	110.9 (2)	C13—O3—C16	117.4 (3)

### Hydrogen-bond geometry (Å, °)

**Table:**

*D*—H···*A*	*D*—H	H···*A*	*D*···*A*	*D*—H···*A*
C5—H5···O1^i^	0.93	2.55	3.303 (4)	139
C16—H16C···O1^ii^	0.96	2.67	3.328 (5)	126
C17—H17B···O4^iii^	0.97	2.55	3.247 (4)	129

Symmetry codes: (i) *x*+1/2, *y*, −*z*+1/2; (ii) *x*+1/2, −*y*+1/2, −*z*+1; (iii) *x*−1/2, *y*, −*z*+1/2.
